# Metastatic testicular tumor presenting as a scrotal hydrocele: An initial manifestation of pancreatic adenocarcinoma

**DOI:** 10.3892/ol.2014.2009

**Published:** 2014-03-28

**Authors:** YEON WOOK KIM, JIN WON KIM, JEE-HYUN KIM, JUNGSIL LEE, EUIJAE LEE, MOON YOUNG KIM, HYUN KYUNG YANG, HYUN CHANG

**Affiliations:** 1 Department of Internal Medicine, Seoul National University Hospital, Seoul, Republic of Korea; 2 Division of Hematology and Medical Oncology, Department of Internal Medicine, Seoul National University Bundang Hospital, Seongnam, Republic of Korea; 3 Department of Pathology, Seoul National University Hospital, Seoul, Republic of Korea; 4 Department of Radiology, Seoul National University Bundang Hospital, Seongnam, Republic of Korea

**Keywords:** adenocarcinoma, neoplasm metastasis, pancreatic neoplasms, testicular hydrocele, testicular neoplasms

## Abstract

Metastatic pancreatic adenocarcinoma involving the testis is a rare condition with a poor prognosis. The current study describes the case of a 69-year-old male who presented with a painful swelling of the left scrotum. Scrotal ultrasonography revealed hydroceles in the scrotal sacs, with the left one being larger in size. The patient underwent left hydrocelectomy and was eventually diagnosed with metastatic adenocarcinoma. Abdominal computed tomography, which was performed to detect the primary cancer, showed a pancreatic tail carcinoma with liver and multiple lymph node metastases, and peritoneal carcinomatosis. The patient received gemcitabine-based chemotherapy but resulted in progressive disease. This case shows that in a patient in whom a primary testicular tumor is unusual due to their age, a testicular mass or hydrocele should be a suspect for possible metastatic disease.

## Introduction

Metastatic testicular tumors that are formed from another solid tumor origin are relatively uncommon. Malignant tumors are known to account for 32.9% of testicular tumors, and metastatic tumors are reported to account for only 6–8% of the malignant tumors (1–2). The most common primary sites reported are the prostate, kidney, lung and gastrointestinal tract (1–3). Only a few cases of metastatic pancreatic carcinoma of the testis have been reported, in which the initial manifestation was a painful scrotal swelling or scrotal mass (3–6).

The present study reports a case of metastatic adenocarcinoma spreading from the pancreas to the testes, which initially presented as a painful scrotal swelling. Written informed consent was obtained from the patient.

## Case report

A 69-year-old male admitted to Seoul National University Bundang Hospital (Seongnam, Korea) presented with a painful swelling in the left scrotum, which was gradually increasing in size. The patient had no history of trauma, no medical history of genitourinary tract anomalies or complaints and did not exhibit any other subjective symptoms.

A physical examination using transillumination revealed a soft, tender mass in the left scrotum and edematous swelling of the surrounding tissue. There were no abnormal findings in the abdomen, chest or extremities. The complete blood cell count, liver function test and serum electrolyte and serum creatinine levels were all within the normal range.

**Figure 1:**
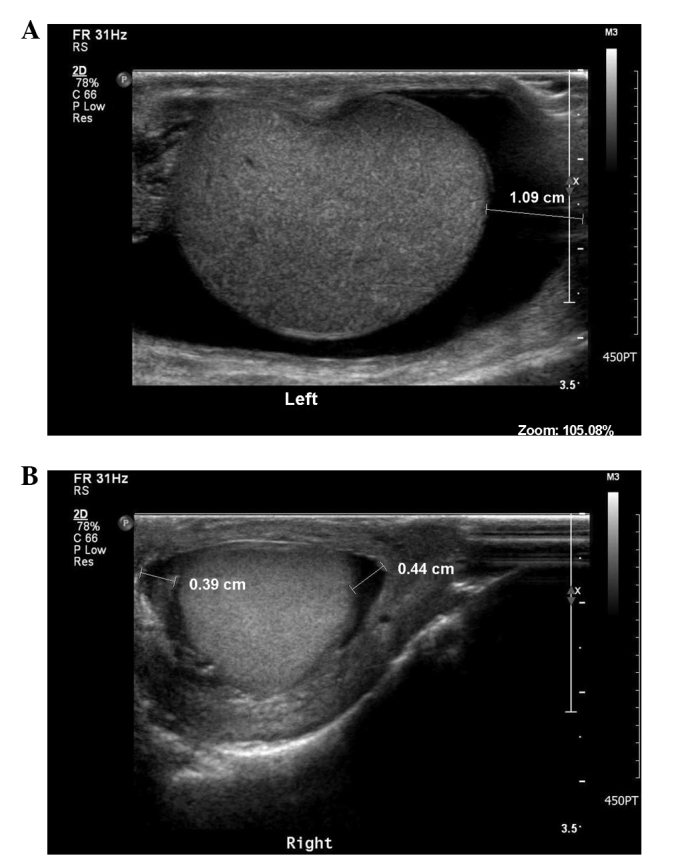
Scrotal ultrasonography findings showing (A) a large amount of fluid collection in the left scrotal sac and (B) a small amount of fluid with internal echogenic material in the right scrotal sac, indicating bilateral hydroceles.

Scrotal ultrasonography showed a large amount of fluid collection in the left scrotal sac, a small amount of fluid with internal echogenic material in the right scrotal sac and diffuse swelling of the scrotal epithelia. The sonographic findings were consistent with a hydrocele of the left testis, a smaller hydrocele of the right testis and associated inflammatory changes in the surrounding soft tissue (Fig. 1).

**Figure 2:**
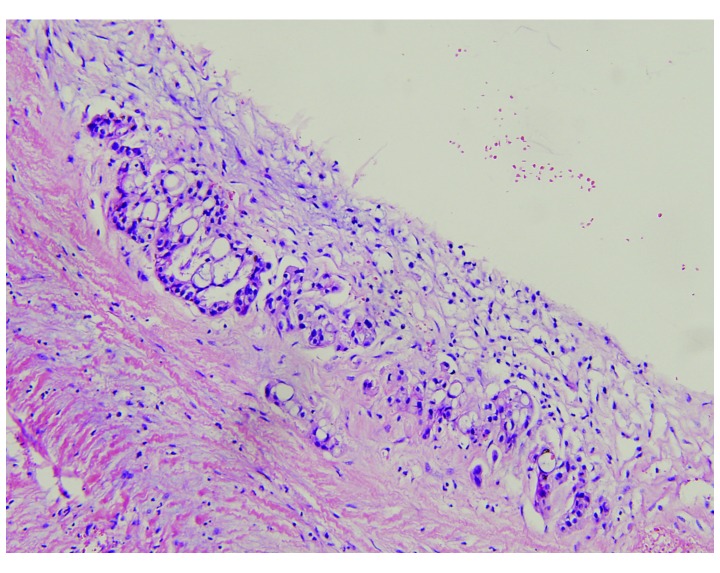
Microscopic findings of the left hydrocele. Localization of adenocarcinoma (hematoxylin and eosin, ×400).

A left hydrocelectomy was performed with the presumptive diagnosis of a benign hydrocele. The pathological diagnosis was consistent with metastatic adenocarcinoma (Fig. 2).

Immunohistochemical staining showed that the tumor cells were positive for cytokeratin 7 and were negative for cytokeratin 20, thyroid transcription factor-1, prostate-specific antigen, estrogen receptor and calretinin.

**Figure 3:**
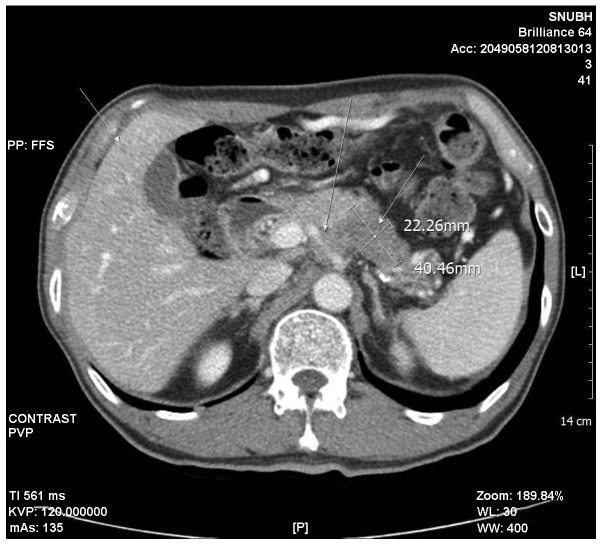
Computed tomography showing a heterogeneous mass in the tail of the pancreas, multiple lymph node enlargements and metastatic nodules in the liver.

Following the diagnosis of metastatic cancer, several examinations were performed to detect the primary cancer. Abdominal-pelvic computerized tomography demonstrated a low-attenuating mass (4.0×2.2 cm) in the pancreatic tail encasing the common hepatic artery. Multiple scattered lesions in the liver, nodular infiltrates in the omentum and peritoneal thickening in the pelvic region were also found, indicating pancreatic tail cancer with liver and multiple lymph node metastasis and peritoneal carcinomatosis (Fig. 3). Whole-body positron emission tomography demonstrated hypermetabolic lesions in the right liver, retropancreatic area, left scrotum and distal pancreas. Tumor marker studies revealed an increased level of cancer antigen 19-9 (4,900 U/ml vs. the normal range of <37 U/ml), whereas the levels of β-human chorionic gonadotropin, lactate dehydrogenase, α-fetoprotein, carcinoembryonic antigen and prostate-specific antigen were in the normal range.

On the basis of these findings, the patient was diagnosed as having pancreatic cancer with metastasis to the liver, peritoneum, lymph nodes and testes. The patient received 3 cycles of gemcitabine-based chemotherapy but resulted in progressive disease. No further chemotherapy was available due to general deconditioning and palliative care was provided.

## Discussion

Metastatic testicular tumors originating from pancreatic cancer are rare. In total, <20 cases have been reported, and it is known that the right testis is more commonly involved (3–6).

Although the current patient first presented with left scrotal swelling without any other symptoms, imaging studies showed hydroceles of the testes, with the hydrocele of the left testis being larger in size. A left hydrocelectomy was performed, as the patient had symptoms only in the left side. However, considering the final diagnosis of metastatic pancreatic cancer and the large extent of the disease, tumor formation in the right testis was suspected also. The treatment plan for the right testis was systemic chemotherapy considering the origin of the metastatic tumor. To the best of our knowledge, this is the first case of metastatic pancreatic adenocarcinoma involving the left and right testes.

Invasion of the testes and paratesticular organs by a metastatic epithelial malignant tumor is rarely the initial clinical manifestation of a primary tumor and may occur as a part of a widely disseminated disease, indicating a poor prognosis with a survival of only 9.1 months from the time of diagnosis (2). The most common primary sites are the prostate, lung, kidney and colon, and less common sites are the stomach, ileum, appendix and pancreas (1–3).

Several routes of metastasis to the testicular tissues have been proposed. These pathways include direct invasion from the contiguous lesion, retrograde venous or arterial embolism, retrograde lymphatic extension from the paraaortic lymph nodes, transperitoneal seeding through a congenital hydrocele and retrograde extension from the vas deferens (1,3,7). Numerous cases appear to reinforce the idea of retrograde lymphatic spread. In the present case, the suspected route of tumor spread was lymphatic and hematogenous, considering metastases of the paraaortic lymph nodes and the liver. However, there was a possibility of transperitoneal seeding through a congenital hydrocele, when considering the painful hydrocele as the first presentation and the extent of the diffuse peritoneal carcinomatosis.

As observed in the current case, metastatic adenocarcinomas of the pancreas, particularly carcinomas of the pancreatic tail, reveal late or no definite clinical symptoms associated with the original tumor. The prognosis of this type of cancer is unfavorable in the majority of cases. In total, <1% of the patients with metastatic pancreatic cancer will survive 5 years following diagnosis (8,9).

The current case indicates that possible metastatic disease should be suspected in patients presenting with a testicular mass or swelling at an age in which a primary testis tumor is unlikely. Such patients should undergo an extensive metastatic evaluation as well as a standard evaluation for a primary testis tumor, particularly if the clinical findings indicate the involvement of other organs (8,10).
